# The Importance of Aortic Valve Bicuspid Phenotype in Valvular Evolution in Pediatric Patients: A Case Report and Literature Mini-Review

**DOI:** 10.3390/ijms241814027

**Published:** 2023-09-13

**Authors:** Amalia Făgărășan, Simona Gurzu, Catalin-Bogdan Satala, Asmaa Carla Hagău

**Affiliations:** 1 Department of Pediatrics III, Faculty of Medicine, George Emil Palade University of Medicine, Pharmacy, Science and Technology of Targu Mures, 540136 Targu Mures, Romania; 2 Department of Pediatric Cardiology, Emergency Institute for Cardiovascular Diseases and Transplantation of Târgu Mureș, 540136 Targu Mures, Romania; 3 Department of Pathology, Clinical County Emergency Hospital, 540136 Targu Mures, Romania; 4 Department of Pathology, George Emil Palade University of Medicine, Pharmacy, Science and Technology of Targu Mures, 540142 Targu Mures, Romania; 5 Doctoral School of Medicine and Pharmacy, I.O.S.U.D., George Emil Palade University of Medicine, Pharmacy, Science and Technology of Targu Mures, 540136 Targu Mures, Romania

**Keywords:** bicuspid aortic valve, pediatric population, aortopathy, calcifications

## Abstract

Bicuspid aortic valve (BAV) is the most commonly encountered congenital malformation in the pediatric population, associated with aortic leaflet degeneration and aortopathy. However, studies on BAV and its complications in children are limited. We present the case of a 16-year-old with type 1B BAV with a raphe with fusion between the right and non-coronary cusps who exhibited severe aortic stenosis, regurgitation, and progressive dilatation of the ascending aorta. Surgical intervention, including aortic valve and aortic root replacement, was performed due to the patient’s deteriorating condition. Histopathological examination revealed degenerative changes and calcifications in the aortic valve and mucoid fibrosis in the ascending aorta. The results are consistent with BAV patients being predisposed to aortic stenosis and regurgitation due to increased mechanical stress and hemodynamic abnormalities. Although more common in adults and a rare complication in pediatric patients, calcification was previously observed concurrently with rapid valve degeneration in our daily practice. Further studies are needed to improve our understanding of the mechanisms underlying BAV-related complications and refine treatment strategies for pediatric patients.

## 1. Introduction

Bicuspidy of the aortic valve (BAV) is the most common congenital malformation in the pediatric population, with a prevalence of almost 0.8% in young adults [1]. It is known that BAV leads to earlier degenerative changes of the aortic leaflets and is associated with aortopathies such as dilatation (with a Z score above 2) and, in severe cases, dissection of the aorta in young adults [2,3]. The rate of aortic dilatation depends on the BAV type. In the pediatric population, BAV type 1B (right-to-non-coronary cusp fusion type) is associated with aortic stenosis or insufficiency with a rapidly progressive evolution and requires early surgical intervention from a young age [4].

Studies regarding the bicuspid aortic valve in the pediatric population and its complications are still scarce, and most studies are still based on the adult population or pediatric aortopathy associated with connective tissue diseases. One particular aspect of the case is the histological assessment of the surgical specimens, which is not routinely performed in such cases, but is extremely useful to understand the pathogenesis of BAV type.

## 2. Case Presentation

A 16-year-old teenager diagnosed in infancy with BAV type 1B presented during follow-up examinations with medium aortic stenosis and progressive dilatation of the ascending aorta with a Z score of +4.21. At the latest admission to our clinic, the patient presented with decreased tolerance for physical activities. A clinical exam revealed a systolic murmur at the aortic point with irradiation at the great vessels of the neck, without jugular venous distension or clinical criteria of Marfan Syndrome and Ehler Danlos. Lung auscultation was normal. The electrocardiogram revealed signs of left ventricle hypertrophy, with an increased Sokolow–Lyon index of 37 mm and Cornell index of 29 mm.

**Figure 1:**
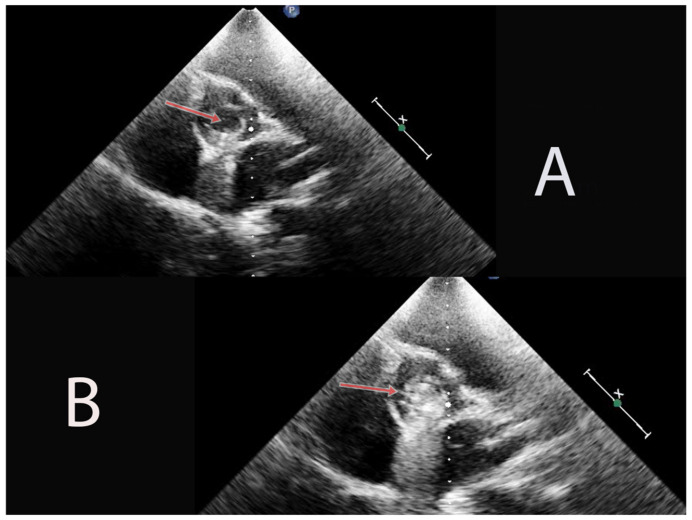
In image (**A**), we can see the aortic valve thickening, with reduced opening during systole (arrow). In image (**B**), evident presence of calcifications within the aortic valve leaflets, contributing to the narrowing of the valve orifice, as revealed by the bright, echo-dense areas with a prominent acoustic shadow (arrow).

The echocardiography exam revealed BAV type 1B with severe aortic stenosis (a mean gradient of 43 mmHg, maximum gradient of 67 mmHg) and medium aortic regurgitation. The aortic leaflets were thickened, with a restrictive opening motion and a significantly reduced orifice of the aortic valve. Also, it showed the presence of calcifications within the aortic valve leaflets that appear as bright, echo-dense areas within the valve structure, indicating the deposition of calcium salts (Figure 1). Furthermore, serial echocardiograms revealed progressive dilatation of the aortic ring, sino-tubular junction and ascending aorta, with significant dilatation during the latest follow-up.

**Figure 2:**
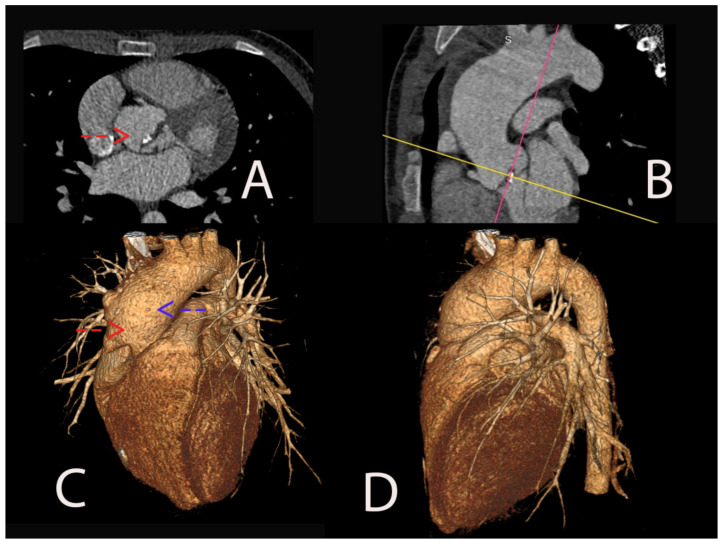
Angiographic computed tomography (CT) image illustrating severe aortic valve stenosis associated with aortic bicuspidy with clear visualization of the aortic cusps and focal valve calcifications at the aortic commissure (red arrow). These calcifications are evident as bright, dense areas within the valve leaflets (**A**). In a sagittal plane, the ascending aorta appears significantly dilated, measuring 4.7 cm in diameter, surpassing the normal range (the colored lines were used as a guide for adjusting imaging planes to the standard cardiac planes- the pink line for the horizontal long axis and the yellow one for the short axis) (**B**). On the three-dimensional (3D) reconstructed angiographic computed tomography (CT) scan, we can see the dilatation of the ascending aorta. Adjacent to the dilated segment, a sacciforme aneurysm is evident (red arrow). Furthermore, the image showcases aortic ulcerations in the right anterior part of the ascending aorta (blue arrow) (**C**,**D**).

Therefore, computed tomography with a contrast substance was performed, which revealed focal calcification at the aortic commissure with important dilatation of the ascending aorta (no = 4.7 cm) with sacciform aneurysm and aortic ulcerations of the right anterior part of the ascending aorta (Figure 2).

**Figure 3:**
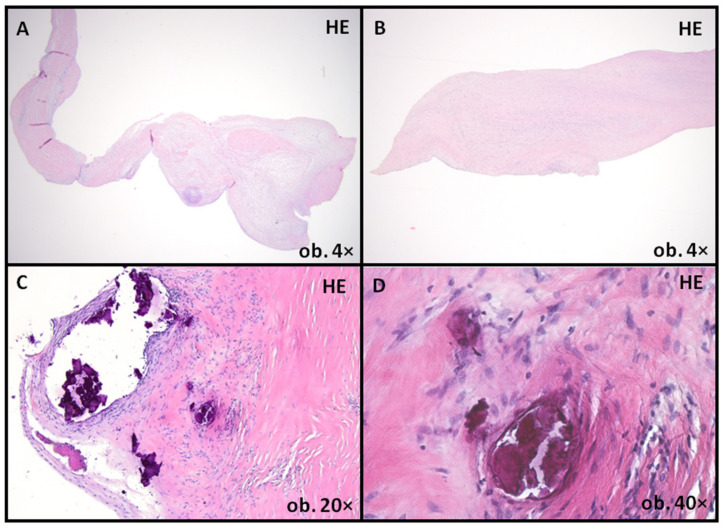
Aortic valve leaflets showing degenerative changes, with architectural distortion (**A**), primarily due to fibrous layer expansion (**B**). Also, multiple calcified foci were present (**C**,**D**). HE: Hematoxylin–Eosin staining.

In this case, due to BAV associated with severe aortic stenosis, according to the European Guidelines of Management of Valvular Diseases, aortic valve surgery was performed to replace the aortic valve with a mechanical valve [5]. Also, concomitant aortic root surgery was performed, with the replacement of the aortic root with a Dacron prosthetic graft. A histopathological examination revealed a fibrotic aortic valve with mucoid and fibro-hyaline degeneration along with calcifications (Figure 3).

**Figure 4:**
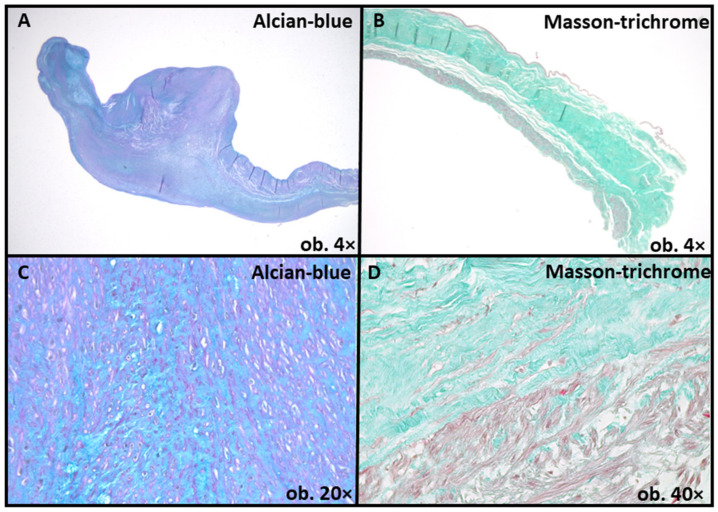
Mucoid accumulation, as emphasized by alcian-blue staining (**A**,**C**) and fibrous layer expansion, as evidenced by Masson-trichrome staining (**B**,**D**).

**Figure 5:**
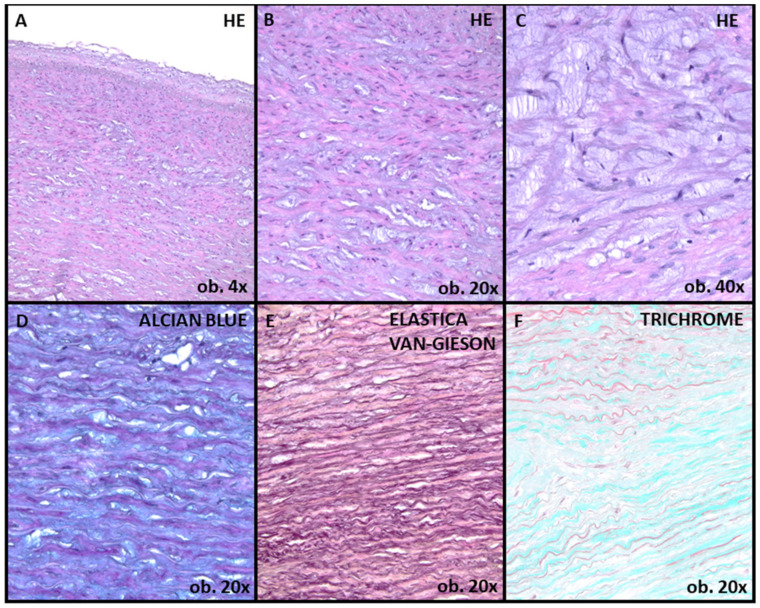
Aortic wall sections showing moderate medial degeneration (**A**,**B**). The mucoid extracellular matrix accumulation is seen, predominantly with intralamellar extension (MEMA-i) (**C**,**D**). Mild elastic fiber fragmentation and collapse, as revealed by van Gieson staining for elastin evidentiation (**E**). Mild medial fibrosis, as revealed by Masson-trichrome staining (**F**). HE: Hematoxylin–Eosin staining.

Furthermore, mucoid accumulation and fibrosis of the media in the ascending aorta wall were assessed (Figure 4 and Figure 5). For this assessment, we used the criteria suggested by the latest Consensus statement on surgical pathology of the aorta: non-inflammatory degenerative diseases-nomenclature and diagnostic criteria, published by the Society for Cardiovascular Pathology and the Association for European Cardiovascular Pathology [6]. Mucoid accumulation was assessed depending on the extension limited to (intralamellar mucoid extracellular matrix accumulation—MEMA-i) or between two adjacent lamellar units (translamellar mucoid extracellular matrix accumulation—MEMA-t). For each staining, standard protocols were used; alcian-blue staining was performed using a 2.5 pH solution method.

## 3. Discussion

BAV is the most common congenital heart defect, with an incidence of almost 0.5–2% in the general population, in which the aortic valve is composed of two leaflets rather than three leaflets [1]. Furthermore, BAV is not only the most common malformation; in many situations, it is seen as a valvulo-aortopathic complex that requires a specific approach in terms of diagnosis and treatment. Therefore, in 2021, Michelena et al. convened to establish an international consensus statement on the nomenclature and classification of BAV and its related aortopathy. This comprehensive review provides a standardized definition of BAV and explores the intricate morphological variations encountered, emphasizing the importance of precise and uniform terminology. Furthermore, it provides a comprehensive assessment of the various aortopathies frequently associated with BAV, including aortic dilation, aneurysm formation and dissection. By providing standardized definitions and classification criteria for these aortic complications, the statement aims to enhance clinical diagnosis, decision-making, and interventional planning [7,8].

### 3.1. Bicuspid Aortic Valve

Morphologically, in clinical practice, the most common type of aortic bicuspid is seen as type 1A (fusion between the left and right cusp) with an incidence of approximately 65%, followed by right cusp fusion with non-coronary cusp-type 2 [9]. Patients with BAV are more prone to present with aortic stenosis because the valve is morphologically stenotic due to restricted mobility. Also, aortic regurgitation is commonly seen.

The complex environment associated with the malformation of the aortic cusps leads to increased endothelial dysfunction and inflammation processes, as well as the remodeling of the extracellular matrix and fibrosis. Chronic inflammation within the valve in BAV patients can trigger the recruitment and activation of immune cells, leading to a vicious cycle of inflammation and calcification. In the acceleration of the calcification process, it seems that the deregulation of the pro-inflammatory and osteo-regulatory signaling pathways plays an important role against the background of increasing mechanical stress [10]. Genotyping studies of families with BAV showed loss-of-function changes R1108X in the Notch 1 extracellular domain responsible for cell proliferation and acceleration of valvular calcification [11]. Despite an increased incidence of right-to-left bicuspid fusion type, calcification of the aortic valve is most seen in patients with right-to-non-coronary cusp fusion type. However, valve calcification is a very rare complication of BAV disease in pediatric patients, with progression mainly in adult patients. In our case, the patient presented with BAV type 1B, severe aortic stenosis, and medium regurgitation, with calcification detected by a computer tomography exam, suggesting a rapid progression of valve degeneration that can occur even in pediatric patients. In terms of the histopathological changes seen on explanted aortic valves, studies have shown that BAV with aortic stenosis or regurgitation had inflammation, neovascularization, and calcium or cholesterol deposits, that were more prevalent in BAV with stenosis compared to BAV with regurgitation, but similar to lesions affecting stenotic tricuspid aortic valves [12].

### 3.2. Aortopathies Associated with BAV

In recent years, it has become evident that BAV is not solely a valvular anomaly, but a complex condition that involves the aortic root and ascending aorta, leading to aortic dilatation and other aortopathies. The phenotype of the aortopathy is determined by the phenotype of the BAV, altered hemodynamics and genetic predisposition. In addition, in pediatric patients with BAV, the fusion between the non-coronary and right-coronary cusps generates progressive lesions over time, including aortopathies, in comparison to adults, in whom there is an overlap with degenerative lesions, regardless of phenotype. Therefore, two main theories were discussed regarding the relationship between BAV and aortic dilatation: the hemodynamic theory and the genetic predisposition.

First, the hemodynamic theory states that increased mechanical stress and significant aortic insufficiency can lead to post-stenotic dilatation. The structurally defective valve leads to a turbulent flow jet within the aortic root, creating abnormal biomechanics, helical flow alterations and uneven wall stress distribution [13]. Histologically, the main mechanisms involved in medial arterial degeneration involve vascular smooth muscle cells (VSMC) apoptosis and different expression of matrix-metalloproteinase and tissue inhibitors of metalloproteinase, leading to abnormal homeostasis of the connective tissue with increased degradation of collagen and elastin [14]. This imbalance also results in excessive degradation of collagen and elastin, leading to aortic valve fibrosis and calcification. Studies have shown that both non-dilated and dilated aortas in patients with BAV present vascular smooth muscle apoptosis. A recent study regarding the histological changes and complications of aortic dilatation and the risk of aortic dissection published by Grewal N et al. revealed the immaturity of the vascular wall due to the lack of differentiated vascular smooth muscle cell (VSMC) and laminin A/C expression [15]. Therefore, in BAV patients, Fibrilin-1, a protein produced by VSMC and essential for the structural stability of the vascular wall, is low.

Also, nitric oxide (NO) is essential for maintaining vascular health and inhibiting calcification processes. Studies based on animal models revealed that NO inhibits the calcification processes in aortic valve cells [16]. In BAV patients, studies have shown that endothelial NO-synthase expression is significantly lower. Therefore, these patients often have reduced NO bioavailability, which may contribute to the intensified fibrosis, and calcification seen in their aortic valves [17]. Also, endothelial dysfunction may also decrease the bioavailability of NO, accelerating the process of vascular remodeling [18].

In addition, Robiseck et al. showed that aortic wall degeneration is absent at birth in patients with isolated BAV compared with patients with BAV and associated congenital diseases, suggesting that aortic wall remodeling increases with age, due to constant turbulent blood flow [19].

Second, there are some studies that emphasize the genetic theory, suggesting that genetic factors may contribute to the development and progression of aortic diseases in individuals with BAV. In a study on a large cohort of pediatric patients with BAV, severe aortic stenosis was one of the strongest predictions of dilatation of the proximal aorta [20]. Also, Grattan et al. showed in the MIBAVA Consortium study that the right to non-coronary fusion was independently associated with ascending aorta dilatation [9]. However, both studies revealed that independently of valve dysfunction, all patients with aortic bicuspid valves presented with higher Z scores for the ascending aorta. Moreover, this theory is supported by some studies that showed an increased incidence of aortic dilatation with tricuspid aortic valves in first-degree relatives of BAV patients [21,22].

It is essential to note that these mechanisms often act in combination, and the precise interplay between them may vary among individuals with BAV, leading to differing degrees of valve calcification, stenosis and aortopathies. Furthermore, it is important to mention that not all patients develop aortic dilatation over time, suggesting the interactions between genetic and hemodynamic factors [13]. Understanding these mechanisms is critical for developing targeted therapies and interventions to prevent or treat aortic valve disease and aortic dilatation in BAV patients. Current guidelines recommend a conservative approach in BAV associated with aortopathy compared with previous recommendations due to a lower incidence of aortic events related to the bicuspid valve populations with aortic diameter below 6 cm [5]. Despite the low incidence of aortic dissection caused by aortic dilatation, it is still a potentially fatal complication. Furthermore, complications are rare in pediatric patients compared to the adult population. For example, in the literature, there were no reported cases of pediatric aortic dissection caused by aortic dilatation and only a few cases of infective endocarditis were reported [23]. However, even though the aortic diameter is currently the major criterion used to predict the optimal time for surgical repair of the ascending aorta, some studies suggest that the aortic diameter is an insufficient predictor of aortic dissection because a significant number of patients present with aortic dissection even though the aortic diameter is below the suggested threshold diameter. For example, according to The International Registry of Acute Aortic Dissection, after a close examination of almost 600 patients with aortic dissection, almost 60% of patients presented with an aortic diameter below 5.5 cm at the time of dissection and 40% of patients had a diameter below 5 cm, suggesting that the aortic diameter may not be the most effective predictor of aortic events [24]. Also, in a recent study published in 2023, Grewal et al. investigated the histopathological features of the ascending aortic wall in pediatric patients (no. 40) with BAV and their implications for future aortopathy. They found that the timing of intimal development in BAV patients differs from that with tricuspid aortic valve: In tricuspid aortic valve patients, the intimal layer expands in the neonatal phase and increases further in thickness until age six, whereas, in BAV patients, intimal thickening starts in early gestation, decreases after birth, and remains thinner throughout life, suggesting that this lack of intimal layer development may be due to a differentiation defect of VSMC and endothelial dysfunction. Furthermore, this study emphasizes the early manifestation of ascending aortic wall pathology in pediatric BAV patients, highlighting the need to consider the pediatric population when searching for predictive markers for future aortopathy [25]. However, in our case, surgical intervention for aortic dilatation was performed due to severe stenosis of the bicuspid valve with a diameter above 4.5 cm of the ascending aorta, as stated in the latest guidelines [5]. Thus, close monitoring and individualized management are crucial in pediatric patients with BAV-related aortopathy.

An important limitation in our case presentation is the absence of genetic testing. As mentioned earlier, BAV often has a genetic foundation, and identifying a specific mutation in the patient could have emphasized their inclusion in higher genetic risk categories. Additionally, exploring the expression of endothelial NO-synthase would have been of interest; however, it did not influence the patient’s outcome. Another significant constraint of the study is our use of a less sensitive staining method for identifying aortic valve calcifications. While Hematoxylin–Eosin staining is indeed effective in revealing prominent calcific centers, it falls short in identifying smaller, punctate calcifications, which could have been detected through the use of von Kossa staining. A more appropriate approach might have involved initially applying von Kossa staining and subsequently counterstaining with Hematoxylin–Eosin. Nevertheless, calcification foci were still identifiable even with the standard staining approach. Moreover, the primary drawback of this case study pertains to its focus on a solitary case report, thereby limiting the generalizability of the findings to the broader population.

However, in our daily clinical practice, we often ponder over important questions: Does the patient’s phenotype have significance? What are the risks involved, and which clinical variable is most crucial in deciding the need for surgical intervention? We emphasize in this case presentation the importance of closely monitoring the phenotype of the patient. Also, we believe that the significance of BAV in pediatric patients and its predictive factors and outcomes are paramount and perhaps even more critical than in adult patients. Understanding and managing BAV in the pediatric population is important due to the potential long-term implications and challenges specific to this age group. Identifying predictive factors for aortopathy and determining optimal interventions are essential for ensuring favorable outcomes and preventing complications in pediatric patients with BAV. As they grow and develop, timely and accurate assessment of BAV-related risks and potential interventions becomes even more vital to providing them with the best possible quality of life and long-term prognosis. Thus, giving utmost attention to BAV in pediatric patients is of utmost importance in the realm of cardiovascular care. Furthermore, we highlight the importance of a multi-disciplinary and intricate approach in the management of pediatric patients with BAV, particularly when determining the optimal timing for intervention.

## 4. Conclusions

In summary, BAV is a complex cardiac anomaly associated with aortopathies that demand specific clinical approaches for diagnosis and treatment. Despite the lower risk of cardiovascular events involving the aorta such as aortic dissection in pediatric patients, aortic dilatation is known as an important risk factor in adults. Therefore, degenerative fibrosis of the ascending aorta could justify aortic replacement in these patients as a preventive measure for cardiovascular events. However, additional studies are required to unravel the underlying mechanisms of aortic pathology in BAV and to refine management strategies for affected individuals, especially in pediatric populations. Lastly, we emphasize the significance of multidisciplinary collaboration among clinicians, surgeons, and researchers to further advance our understanding of BAV and its associated aortopathies.

## Data Availability

No new data were created or analyzed in this study. Data sharing is not applicable to this article.
